# Metabolomic Profiles During and After a Hypertensive Disorder of Pregnancy: The EPOCH Study

**DOI:** 10.3390/ijms26136150

**Published:** 2025-06-26

**Authors:** Mark A. Hlatky, Chi-Hung Shu, Nasim Bararpour, Brenna M. Murphy, Sabina M. Sorondo, Nicholas J. Leeper, Frank Wong, David K. Stevenson, Gary M. Shaw, Marcia L. Stefanick, Heather A. Boyd, Mads Melbye, Oshra Sedan, Ronald J. Wong, Michael P. Snyder, Nima Aghaeepour, Virginia D. Winn

**Affiliations:** 1 Department of Health Policy, Stanford University School of Medicine, Stanford, CA 94305, USA; 2 Department of Medicine, Stanford University School of Medicine, Stanford, CA 94305, USAstefanick@stanford.edu (M.L.S.); 3 Department of Anesthesia, Stanford University School of Medicine, Stanford, CA 94305, USA; chshu@stanford.edu (C.-H.S.); naghaeep@stanford.edu (N.A.); 4 Department of Genetics, Stanford University School of Medicine, Stanford, CA 94305, USAfhswong@stanford.edu (F.W.); mpsnyder@stanford.edu (M.P.S.); 5 Department of Surgery, Stanford University School of Medicine, Stanford, CA 94305, USA; 6 Department of Pediatrics, Stanford University School of Medicine, Stanford, CA 94305, USA; dks750@stanford.edu (D.K.S.); gmshaw@stanford.edu (G.M.S.); rjwong@stanford.edu (R.J.W.); 7 Department of Obstetrics and Gynecology, Stanford University School of Medicine, Stanford, CA 94305, USA; vwinn@stanford.edu; 8 Department of Epidemiology Research, Statens Serum Institut, Copenhagen 2300, Denmark; hoy@ssi.dk; 9 Danish Cancer Institute, and Department of Clinical Medicine, University of Copenhagen, Copenhagen 2200, Denmark; melbye@cancer.dk

**Keywords:** preeclampsia, gestational hypertension, metabolomics, machine learning, case–control study

## Abstract

**:** Hypertensive disorders of pregnancy are associated with a higher risk of later cardiovascular disease, but the mechanistic links are unknown. We recruited two groups of women, one during pregnancy and another at least two years after delivery, including both cases (with a hypertensive disorder of pregnancy) and controls (with a normotensive pregnancy). We measured metabolites using liquid chromatography–mass spectroscopy and applied machine learning to identify metabolomic signatures at three time points: antepartum, postpartum, and mid-life. The mean ages of the pregnancy cohort (58 cases, 46 controls) and the mid-life group (71 cases, 74 controls) were 33.8 and 40.8 years, respectively. The levels of 157 metabolites differed significantly between the cases and the controls antepartum, including 19 acylcarnitines, 12 gonadal steroids, 11 glycerophospholipids, nine fatty acids, six vitamin D metabolites, and four corticosteroids. The machine learning model developed using all antepartum metabolite levels discriminated well between the cases and the controls antepartum (c-index = 0.96), postpartum (c-index = 0.63), and in mid-life (c-index = 0.60). Levels of 10,20-dihydroxyeicosanoic acid best distinguished the cases from the controls both antepartum and postpartum. These data suggest that the pattern of differences in metabolites found antepartum continues to distinguish women who had a hypertensive disorder of pregnancy from women with a normotensive pregnancy for years after delivery.

## 1. Introduction

Hypertensive disorders of pregnancy (HDPs), which include preeclampsia and gestational hypertension, are associated with an increased risk to both the mother and the fetus [1]. HDPs resolve clinically soon after delivery, and have long been regarded as having no long-term adverse consequences. Recent studies, however, have shown that women with a history of an HDP have an elevated risk of developing cardiovascular disease (CVD) later in life, particularly chronic hypertension and atherosclerotic CVD [2,3,4,5]. The potential pathophysiologic links between HDPs in early life and CVD in later life are unknown. We designed the EPOCH (Effect of Preeclampsia on Cardiovascular Health) study to search for biomarkers that could provide insights into why HDPs are associated with the later development of CVD [6,7].

Metabolic changes have long been associated with the late development of CVD. Alterations in lipid metabolism (hypercholesterolemia) and glucose metabolism (diabetes), and chronic kidney disease are well-established risk factors for the development of atherosclerotic CVD. Newer biomarker assays, such as the small molecules assessed by metabolomics, can now be used to assess CVD risk and pathophysiology [8]. An increased risk of atherosclerotic CVD has been associated with several metabolomic changes, most notably higher levels of branch-chain amino acids (leucine, isoleucine, and valine), phenylalanine, monosaturated fatty acids, acylcarnitines, and trimethylamine N-oxide (TMAO) [8]. Levels of branch-chain amino acids have also been associated with insulin resistance and type 2 diabetes, while levels of alanine, hippuric acid, and hexadecanedioate have been associated with blood pressure changes [8].

A normal pregnancy induces a wide array of metabolic adaptations in the mother [9]. Superimposed upon these normal adaptations of pregnancy are metabolic alterations due to an HDP. It is uncertain, however, whether the metabolic changes in a mother with an HDP persist following delivery, or are related to the later development of CVD. In this study we aimed to characterize the metabolic profiles associated with HDPs in the participants of the EPOCH study at three time points: during pregnancy (antepartum), a few weeks after delivery (postpartum), and several years after delivery (mid-life), and identify the metabolic pathways most affected by HDPs.

## 2. Results

### 2.1. Study Participants

**Figure 1:**
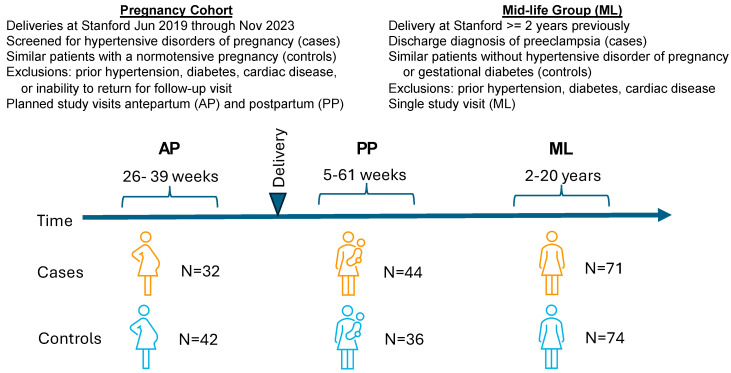
Study overview: Study participants were enrolled in two groups. The pregnancy cohort (left panel) was invited to make two study visits, one antepartum (AP) and one postpartum (PP). The mid-life (ML) group was invited to make a single study visit.

We enrolled participants in two groups, one during pregnancy and another in mid-life, several years after pregnancy (Figure 1). The mean age was 33.2 years in the pregnancy cohort and 40.8 years in the mid-life group, and most participants were White or Asian (Table 1). The cases were more likely than the controls to have higher blood pressures, higher body mass indexes, and to have delivered at an earlier gestational age (Table 1).

At the antepartum visit, 27 cases (85%) had preeclampsia and five cases had gestational hypertension. At the postpartum visit, 34 cases (77%) had prior preeclampsia, and ten cases had prior gestational hypertension. Only 17 cases and 32 controls were able to complete both the antepartum and postpartum visits, but metabolite profiles were measured in all participants (Table 1).

### 2.2. Antepartum Comparisons

**Figure 2:**
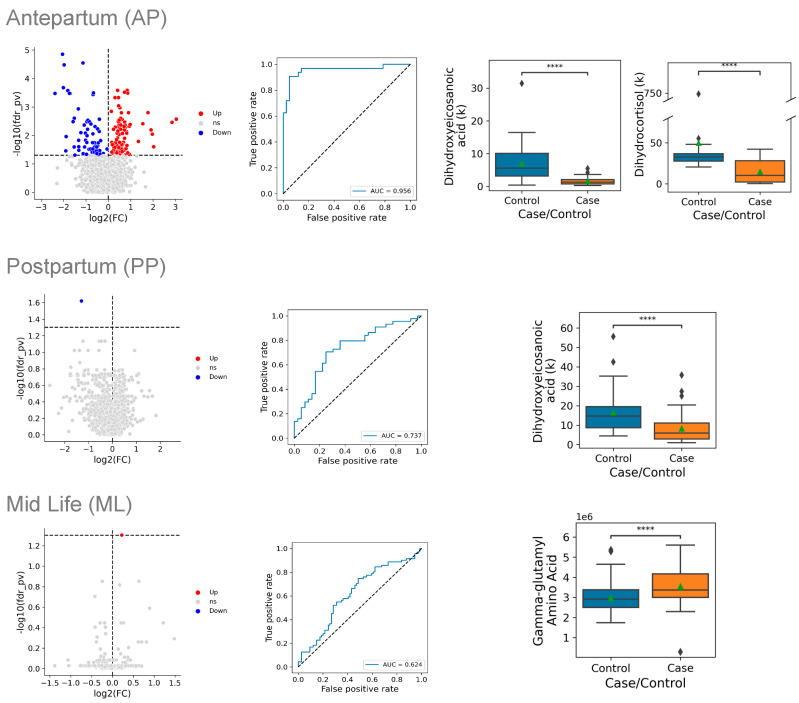
Metabolite levels of cases vs. controls at three time points during and after pregnancy. Data are presented at three time points: antepartum (AP) on the top row, postpartum (PP) in the middle row, and mid-life (ML) in the bottom row. Within each row, the left panel displays the natural log of the fold-change in levels between cases and controls, plotted on the horizontal axis, and the adjusted *p*-value, plotted on the vertical axis (log scale); the dashed horizontal line indicates an adjusted *p*-value of 0.05. Red dots indicate that the metabolite level is significantly higher in cases and blue dots indicate that the metabolite level is significantly lower in cases. The center panel of each row displays the receiver operating characteristic (ROC) curves for the multivariate model of the metabolite signature that distinguished cases from controls. The horizontal axis shows the false-positive rate (1-specificity) of the multivariate model, while the vertical axis indicates its sensitivity. The right panel(s) of each row display box-and-whisker plots of the most discriminating metabolites between cases and controls. The top line of the box is the 75th percentile, the bottom line is the 25th percentile, and the line in the box is the median. The point indicates the mean value in each group **** = < 0.0001.

**Figure 3:**
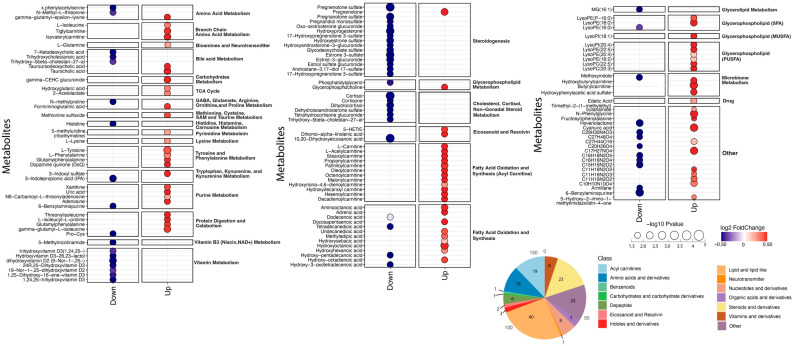
Antepartum metabolites that differ between cases and controls. Each line displays a single metabolite, labeled on the left, with blue dots indicating lower levels in cases than in controls, while red dots indicate higher levels in cases than controls. The size of the dots is proportional to the −log10 *p*-value, and the color indicates the log2 fold change between cases and controls. The metabolites are grouped by metabolic pathway, which is labeled on the right. The numbers of metabolites in various classes are summarized in the lower right corner.

Among the total of the 1987 analytes measured, cases with an HDP differed significantly from the controls in the levels of 157 analytes (8%), 93 of which were higher in the cases, and 64 of which were lower in the cases (Figure 2). The metabolites that differed significantly between the cases and controls fell into several classes and pathways (Figure 3). Levels of 33 distinct metabolites related to fatty acid oxidation and synthesis were increased in the cases, including 19 acylcarnitines and 9 fatty acids; in addition, 11 metabolites in the glycerophospholipid pathway were increased in the cases. Levels of 12 gonadal steroids were reduced in the cases compared with the controls, as were levels of 6 vitamin D metabolites, and 4 corticosteroids (Figure 3).

The metabolites that differed most significantly between the cases and the controls antepartum included 10,20-dihydroxyeicosanoic acid (decreased 76%, *p* = 0.00001), cortisol (decreased 75%, *p* = 0.00003), and pregnenolone sulfate (decreased 54%, *p* = 0.00003) (Table 2). A multivariate model based on the full set of metabolites had a c-index (area under the receiver operating curve) of 0.956, indicating excellent discrimination between the cases and the controls. A parsimonious model that used two metabolites as predictors provided a similar level of discrimination.

### 2.3. Postpartum Comparisons

Postpartum, cases with an HDP differed significantly from the controls only in the levels of a single metabolite, 10,20-dihydroxyeicosanoic acid, which was reduced by 59% in the cases compared with the controls (*p* = 0.024), which is close to the 76% reduction found antepartum (*p* = 0.00001). A multivariate model developed using all postpartum metabolite levels had very good discrimination, with a c-index of 0.737. A parsimonious model that used a single predictor discriminated between the cases and the controls equally well. 

### 2.4. Mid-Life Comparisons

In the mid-life samples, cases with an HDP differed significantly from the controls in their levels of a single metabolite, gamma-glutamyl threonine, with levels that were 17% higher in the cases (*p* = 0.0497). The levels of gamma-glutamyl threonine did not differ significantly between the cases and controls in the antepartum samples, but several related metabolites did, including gamma-glutamyl isoleucine (Table 2), gamma-glutamyl leucine, and gamma-glutamyl-epsilon-lysine (Supplemental Table S1). A multivariate model developed using all mid-life metabolite levels showed moderate discrimination, with a c-index of 0.624, and a parsimonious model based on a single predictor had an equivalent level of discrimination.

### 2.5. Temporal Analyses

**Figure 4:**
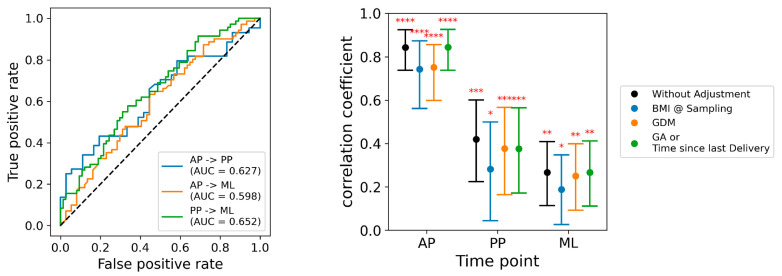
Multivariate models applied at subsequent time points, and adjusted for baseline covariates. **Left panel**: Discrimination of models applied at different time points, displayed as AUCs. The blue line indicates the discrimination of the model developed with the antepartum samples when applied to the postpartum samples (AUC = 0.63), and to the mid-life samples (AUC = 0.60). The orange line indicates the discrimination of the model developed in the postpartum samples when applied to the mid-life samples (AUC = 0.65). **Right panel**: Effect of adjustment for covariates on the performance of the postpartum model. The vertical axis indicates the partial correlation coefficient, and the horizontal axis indicates the results at three study time points: antepartum (AP), postpartum (PP), and mid-life (ML). The black points indicate the performance of the unadjusted model, and the error bars indicate its 95% confidence limits. Model performance adjusted for body mass index (BMI) is shown with blue symbols, model performance adjusted for gestational diabetes (GDM) is shown with orange symbols, and model performance is adjusted for study visit timing (weeks of gestational age (GA) antepartum, and weeks after delivery postpartum and mid-life) is shown in green symbols. Statistical significance levels indicated as: 0.01< * = <0.05; 0.001< ** = < 0.01; 0.0001< *** = < 0.001; **** = < 0.0001.

The signature developed using a multivariate model of all antepartum metabolite levels also discriminated between the cases and the controls when it was applied to the metabolite profiles in the postpartum samples (c-index of 0.627), and in the independent group of mid-life participants (c-index of 0.598) (Figure 4). The postpartum model discriminated between the cases and the controls when applied to the mid-life samples, with a c-index of 0.652. 

### 2.6. Covariates

Cases were more likely than the controls to have a higher body mass index, a longer interval between delivery and the postpartum visit, and gestational diabetes in the index pregnancy (Table 1). Adjustment for body mass index attenuated the correlation coefficients of the predictive models somewhat, but all correlations remained statistically significant (Figure 4). Model discrimination was only slightly reduced by adjustment for gestational diabetes mellitus, and essentially unaffected by adjustment for the timing of the study visit (Figure 4).

Twenty-three cases (72%) were treated with betamethasone at a median of 4 days (interquartile range, 1.5 to 9.5 days) prior to the antepartum study visit, while no controls received betamethasone. Antepartum levels of plasma corticosteroids were lower in the cases treated with betamethasone than in the untreated cases, and levels were significantly correlated with the time since betamethasone treatment (Supplemental Figure S1).

## 3. Discussion

In this study, we found widespread differences antepartum in the plasma metabolomes of women who had a new-onset hypertensive disorder of pregnancy and women who did not (Figure 2 and Figure 3). Almost all of the differences in the levels of individual metabolites resolved postpartum, and only the levels of 10,20-dihydroxyeicosanoic acid differed significantly postpartum. Nevertheless, the metabolic signature that we identified antepartum (i.e., the overall pattern of differences found by analysis of all metabolite levels antepartum) could distinguish the cases from the controls when the model was applied to metabolite levels measured at the postpartum and mid-life time points, suggesting that the metabolic disturbances at the height of the illness may persist for prolonged periods of time after the clinical resolution of an HDP.

The metabolites that differed significantly antepartum between the cases and controls include many that are of biological interest (Figure 3). A total of 33 distinct metabolites related to fatty acid oxidation differed, almost all of which were significantly increased in the cases (Figure 3). Levels of 12 gonadal steroids were reduced in the cases, as were levels of 4 corticosteroids. Several exogenous molecules also differed between cases and controls, underscoring the potential influence of diet, gut microbiota, and environmental exposures on metabolic alterations in women with an HDP.

Levels of 10,20-dihydroxyeicosanoic acid were significantly reduced in the cases compared with the controls both antepartum and postpartum, and were the strongest features that best distinguished the two groups at both time points. The biologic significance of this molecule is uncertain, but it is structurally similar to the dihydroxyeicosatrienoic acids, which are derived from the vasoactive epoxyeicosatrienoic acids by the action of soluble epoxide hydrolase [10]. The potential role of these metabolites in the pathogenesis of an HDP and subsequent CVD warrants further investigation.

We also found significantly lower antepartum levels of four corticosteroids in the cases compared with the controls, and cortisol was one of the three strongest predictors that distinguished the cases from the controls antepartum (Table 2). Cases with preeclampsia were more likely than the controls, however, to have been treated with betamethasone because of their higher risk of preterm birth, and the lower corticosteroid levels appear to be due to the effects of this medication rather than the underlying pathophysiology of preeclampsia. Corticosteroid levels could also be affected by altered hypothalamic–pituitary–adrenal axis function or increased activity of placental 11β-hydroxysteroid dehydrogenase type 2 due to an HDP. Similar reductions in cortisol levels have been reported by the authors of other metabolomic studies of preeclampsia [11,12].

We also identified novel associations between gut-microbiota-derived metabolites and HDP risk. Indole-3-propionic acid (IPA), a microbial tryptophan metabolite with antioxidant and anti-inflammatory properties, was reduced by 53% in the cases compared with the controls (*p* = 0.004, Supplemental Table S1). In light of the emerging evidence linking gut dysbiosis to HDPs, these findings suggest the need for further investigation into microbiome-targeted interventions (e.g., probiotics, dietary modifications) that might mitigate preeclampsia-associated metabolic changes [13,14].

We previously examined the proteomic profiles of these same EPOCH study participants, and identified differences in the levels of 28 proteins between the cases and controls that persisted from postpartum to mid-life, including five from the complement cascade [7]. Interestingly, the alterations in levels of individual proteins that we found in these EPOCH participants were more widespread and persistent than the metabolomic differences we identified in the present study: we found significant differences in the postpartum levels of 189 proteins versus one metabolite, and in the mid-life levels of 224 proteins versus one metabolite. It is unclear as to why we found so many fewer differences in plasma metabolites than in plasma proteins. One possibility is that the changes in protein levels were quantitatively greater antepartum than the changes in metabolite levels. Another possibility is that changes in the levels of plasma proteins are more closely related to the underlying biology of an HDP, and that changes in the levels of metabolites are more distal in the disease process. Nevertheless, the patterns of differences we found in the proteome and in the metabolome continued to discriminate between the cases and the controls at subsequent time points.

### 3.1. Prior Studies

Our findings are largely consistent with those of prior studies examining the antepartum metabolomic changes associated with an HDP. Bartho and associates enrolled 50 women with early-onset preeclampsia and 25 gestational-age-matched controls at a mean gestational age of 29 weeks, and measured metabolites using an LC–MS system [15]. A total of 90 of the 174 metabolites assayed differed significantly between women with preeclampsia and the controls, with the largest differences being in L-cystine, L-cysteine, L-acetylcarnitine, carnitine, and D-alpha-aminobutyric acid. The pathways that were most altered in their study included aminoacyl-tRNA biosynthesis; arginine biosynthesis; D-glutamine and D-glutamate metabolism; valine, leucine, and isoleucine biosynthesis; and the biosynthesis of unsaturated fatty acids. Their findings align with those of our study, particularly the elevations in acylcarnitines and amino acids, as well as the significant reduction in cortisol levels in the cases (Table 2).

Yao and associates reviewed 41 metabolomics studies of preeclampsia during pregnancy that were published between January 2020 and November 2021 [16]. Most of these 41 studies used mass spectroscopy (27), as did we in the present study, while 14 used nuclear magnetic resonance to identify metabolites; 23 studies used an untargeted approach, as did we in the present study, while 18 studies were targeted. The 33 metabolites that were most frequently reported as being significantly altered in preeclampsia included creatinine, glycine, L-isoleucine, and glucose (each in seven studies); L-phenylalanine (six studies); and decanoylcarnitine, L-carnitine, creatine, histidine, L-leucine, 3-hydroxybutyric acid, L-valine, L-threonine, and acetic acid (each in five studies) [16]. The pathways that were most frequently implicated included amino acid metabolism (eight studies), carbohydrate metabolism (three studies), aminoacyl-tRNA biosynthesis, and sphingolipid metabolism. These findings align with our results, particularly regarding branched-chain amino acid dysregulation (leucine), aromatic amino acid alterations (phenylalanine), and lipid metabolism perturbations (acylcarnitines and lysophospholipids).

Few previous studies have examined whether there are postpartum metabolomic differences in women who developed an HDP. Hong and colleagues compared metabolomic profiles in women with preeclampsia who delivered preterm with controls who delivered at term, analyzing maternal blood collected between 24 and 72 h post-delivery [17]. They used LC–MS to measure 380 metabolites, 47 of which differed between the 79 women with preeclampsia and preterm birth and the 980 healthy controls who had a term delivery. They identified differences in seven amino acids, four acylcarnitines, ten diacyl- or triacylglycerols, five cholesterol esters, and two steroids, including cortisol [17]. Their findings complement our results, particularly regarding amino acid and acylcarnitine dysregulation in HDP. However, their postpartum samples were collected within one to three days post-delivery, and may still reflect the metabolic state during pregnancy, whereas we collected postpartum samples much later—at least six weeks after delivery (median of ten weeks).

Additionally, several studies have evaluated whether metabolomic markers measured in early pregnancy might predict the subsequent development of preeclampsia. While plasma metabolic markers have less predictive accuracy than other biomarkers [18], one study found that elevations in plasma acylcarnitines during the first trimester were associated with the later development of preeclampsia [19]. This supports the hypothesis that metabolic dysregulation in an HDP begins early in gestation and persists throughout pregnancy, with potential long-term cardiovascular consequences.

### 3.2. Limitations

This study has several limitations. We cannot determine whether the observed changes in any given metabolite are due to the underlying disease process or other factors, such as medications, diet, and gut microbiota. Study recruitment and follow-up were impacted by the COVID-19 pandemic, which limited the ability of some participants to complete both antepartum and postpartum study visits. Additionally, our study population was drawn from a single medical center, and most participants were Hispanic, Asian, or White, potentially limiting the generalizability to broader populations. Finally, not all identified eligible patients were enrolled, which may have influenced the comparisons between the cases and controls, particularly in the mid-life group. Replication of our findings in other cohorts is essential for validating these results.

### 3.3. Conclusions

We found substantial antepartum differences in the plasma metabolomes of women with a hypertensive disorder of pregnancy compared with women with a normotensive pregnancy. Metabolomic alterations were widespread during pregnancy, but the levels of most individual metabolites were no longer significantly different after delivery. Nevertheless, the pattern of changes that we identified in the plasma metabolome using machine learning continued to distinguish the cases from controls both postpartum and in mid-life, underscoring the potential for metabolic alterations during pregnancy to contribute to long-term cardiovascular risk in women. Future studies should investigate whether and how early metabolic shifts during pregnancy contribute to later CVD risk, and explore novel therapeutic strategies to mitigate metabolic dysfunction in preeclampsia.

## 4. Materials and Methods

### 4.1. Study Design

The design of the EPOCH study and the baseline characteristics of study participants have been described previously [6,7]. Briefly, we enrolled two groups of participants, one during pregnancy, and a second that was two or more years after pregnancy. In each group, we enrolled participants with either a new-onset HDP (cases) or a normotensive pregnancy (controls) (Figure 1).

For the pregnancy cohort, we prospectively screened women receiving obstetrical care at Stanford University Medical Center for patients with an HDP and identified demographically similar women with a normotensive pregnancy. We enrolled women aged between 18 and 45 years and excluded women who were unable to return for a postpartum visit, as well as those with either chronic hypertension, diabetes mellitus, heart disease, chronic kidney disease, autoimmune disease, or cancer prior to pregnancy. We defined preeclampsia, gestational hypertension, and their severity using the 2013 criteria of the American College of Obstetricians and Gynecologists [1], which require blood pressure readings of ≥140 mm Hg systolic or ≥90 mm Hg diastolic on two occasions at least four hours apart after 20 weeks of gestation in a woman with a previously normal blood pressure. We defined a normotensive pregnancy as one without preeclampsia, gestational hypertension, and gestational diabetes. We invited participants in the pregnancy cohort to attend two study visits: the first was antepartum, after 24 weeks’ gestational age, and the second was at least six weeks postpartum.

For the mid-life group, we searched the electronic health records of Stanford Health Care to identify women aged between 21 and 55 years who had a pregnancy complicated by preeclampsia at least two years earlier, who delivered at Stanford, were premenopausal, and met the inclusion and exclusion criteria described above. We contacted potentially eligible subjects to invite them to participate in the study and complete a single clinical research visit. We identified control subjects for each case who were frequency-matched based on their maternal age, time since delivery, race, and ethnicity, and invited them to participate using the methods described above. 

This study was approved by the Stanford Institutional Review Board, and all participants provided written informed consent.

### 4.2. Sample Collection

During each study visit, we drew fasting whole-blood samples into ethylenediaminetetraacetic-acid-containing vacutainers, which we inverted immediately and placed on ice. We centrifuged samples for 30 min at 4 °C at 1500× *g*, transferred the supernatant (avoiding the pellet) into fresh 2 mL microfuge tubes, and centrifuged them at 12,100× *g* for one minute at 4 °C. We then aliquoted the supernatant into cryotubes and stored them at −80 °C. We processed all of the samples within 60 min of blood collection.

### 4.3. Metabolomic Assays

We extracted metabolites and complex lipids in a 96-well high-throughput fashion using biphasic separation with cold methyl tert-butyl ether, methanol, and water. We first added 1 mL of methyl tert-butyl ether to 40 μL of plasma, spiked the sample with 40 μL of deuterated lipid internal standards (Sciex, Framingham, MA, USA: cat# 5040156, lot# LPISTDKIT-103), then agitated the samples at 4 °C for 30 min. We then added 250 μL of cold water, vortexed samples for 1 min, then centrifuged them at 3800× *g* for 5 min at 4 °C to yield an upper organic phase containing lipids and a lower aqueous phase containing metabolites, with precipitated proteins at the bottom of the tube. For quality control, reference plasma samples (40 μL plasma), as well as controls lacking samples (blanks), were processed in parallel.

To further precipitate proteins, we added 500 μL of 1:1:1 acetone/acetonitrile/methanol spiked with 15 labeled metabolite internal standards to 300 μL of the aqueous phase and incubated these overnight at −20 °C. After centrifugation at 3800× *g* for 10 min at 4 °C, we dried the metabolic extracts under a stream of nitrogen gas and resuspended the samples in 100 μL 50/50 methanol/water for liquid chromatography–mass spectroscopy (LC–MS).

We analyzed the metabolic extracts four times using hydrophilic interaction liquid chromatography (HILIC) and reverse-phase liquid chromatography (RPLC) separation in both positive and negative ionization modes to maximize the number of identified metabolites [20]. We acquired data on a Thermo Q Exactive HF mass spectrometer for HILIC (Thermo Fisher Scientific, Bremen, Germany) and a Thermo Q Exactive Plus mass spectrometer for RPLC (Thermo Fisher Scientific, Bremen, Germany). Both instruments were equipped with a heated electrospray ionization (HESI-II) probe and operated in the full MS scan mode. MS/MS data were acquired on quality control samples consisting of an equimolar mixture of all samples in the study. We performed HILIC experiments using a ZIC-HILIC column 2.1 × 100 mm, 3.5 μm, 200 Å (Merck Millipore, Darmstadt, Germany) and mobile-phase solvents consisting of 10 mM ammonium acetate in 50/50 acetonitrile/water (A) and 10 mM ammonium acetate in 95/5 acetonitrile/water (B). We performed RPLC experiments using a Zorbax SBaq column 2.1 × 50 mm, 1.7 μm, 100 Å (Agilent Technologies, Palo Alto, CA, USA) and mobile-phase solvents consisting of 0.06% acetic acid in water (A) and 0.06% acetic acid in methanol

We ensured data quality by (i) injecting 6 and 12 pool samples to equilibrate the LC–MS system prior to running the sequence for RPLC and HILIC, respectively, (ii) injecting a pooled sample every 10 injections to control for signal deviation with time, and (iii) checking the mass accuracy, retention time, and peak shape of internal standards in each sample.

We analyzed data from each mode independently using Progenesis QI software (v2.3, Nonlinear Dynamics, Durham, NC, USA). We discarded metabolic features from blanks and those that did not show sufficient linearity upon dilution in the quality control samples (r < 0.6). We retained for further analysis only metabolic features that were present in more than two-thirds of the samples. We imputed missing values by drawing from a random distribution of low values in the corresponding sample and corrected for intensity drift using Systematical Error Removal using Random Forest. We verified data quality post-normalization by ensuring the clustering of pooled sample replicates on a principal component analysis plot.

We merged data from each mode and annotated metabolic features. We annotated peaks first by matching the experimental *m*/*z*, retention time, and MS/MS spectra to an in-house library of analytical-grade standards. We identified the remaining peaks by matching the experimental m/z and fragmentation spectra to publicly available databases, including the Human Metabolome Database and MassBank of North America (MoNA) using the R package ‘metID’ (v0.2.0). We used the Metabolomics Standards Initiative level of confidence to grade the metabolite annotation confidence (level 1 to level 3). Level 1 represents formal identifications where the biological signal matches the accurate mass, retention time, and fragmentation spectra of an authentic standard run on the same platform. For level 2 identification, the biological signal matches the accurate mass and fragmentation spectra available in one of the public databases listed above. Level 3 represents putative identifications that provide the most likely name based on previous knowledge.

### 4.4. Case–Control Comparisons

We compared metabolite levels between the controls and cases at each time point, with differences expressed in terms of the fold-change (FC):

log_2_(FC) = mean(log_2_(case)) − mean(log_2_(control))

We assessed the statistical significance of the differences in metabolite levels with Mann–Whitney U tests and applied the Benjamini–Hochberg method [21] to adjust for multiple hypothesis testing, set the false discovery rate to 0.05, and adjust the raw *p*-values.

### 4.5. Multivariate Modeling

We used multivariate models based on differences in metabolite levels to distinguish the cases from controls at each time period, using gradient-boosted trees (XGBoost) with repeated 10-fold cross-validation [22]. We averaged the results of these iterations, and measured the final model discrimination as the area under the receiver operating characteristic curve (c-index).

We developed parsimonious predictions that used fewer metabolite levels as predictors by using additional, retrained XGBoost models, in which we systematically varied the subsets of predictors (i.e., metabolite levels), in a descending order of their importance in the original model. Starting with an initial model that included the most important predictor from the original model, we added predictors incrementally, computed Mann–Whitney U *p*-values for each additional model, compared these *p*-values with the *p*-value of the original model, and identified the most parsimonious model (i.e., the model including the fewest metabolites) that provided the same level of statistical significance as the original model.

### 4.6. Temporal Analyses

We tested the ability of the individual multivariate models developed at each study time point to discriminate between the cases and controls at other time points by applying them to data from samples collected at later time points. Specifically, we tested the antepartum model by applying it to the data from the postpartum samples, and tested the postpartum model by applying it to the data from the independent mid-life group.

## Figures and Tables

**Table 1:** Baseline characteristics, by case–control status and study visit.

	Antepartum Visit			Postpartum Visit			Mid-Life Visit		
	Cases (n = 32)	Controls (n = 42)	*p*- Value	Cases (n = 44)	Controls (n = 36)	*p*-Value	Cases (n = 71)	Controls (n = 74)	*p*-Value
Clinical characteristics									
Race *									
White	12 (38%)	25 (60%)	0.10	22 (50%)	23 (64%)	0.31	38 (54%)	45 (61%)	0.47
Asian/Pacific Islander	7 (22%)	12 (29%)	0.70	10 (23%)	10 (28%)	0.80	17 (24%)	25 (34%)	0.26
Black/African American	1 (3%)	2 (5%)	1.00	1 (2%)	2 (6%)	0.59	3 (4%)	3 (4%)	1.00
Native American/Alaskan	0 (0%)	0 (0%)	1.00	1 (2%)	0 (0%)	1.00	1 (1%)	2 (3%)	1.00
Not reported	12 (38%)	5 (12%)	0.005	11 (25%)	2 (6%)	0.03	16 (23%)	8 (11%)	0.09
Hispanic ethnicity	9 (28%)	6 (14%)	0.14	14 (32%)	4 (11%)	0.03	19 (27%)	14 (19%)	0.26
Gestational diabetes	8 (25%)	0 (0%)	0.0004	9 (20%)	0 (0%)	0.003	6 (8%)	0 (0%)	0.01
Preterm HDP onset (<37 weeks)	31 (97%)	0 (0%)	NA	25 (57%)	0 (0%)	NA	31 (44%)	0 (0%)	NA
Gestational age at delivery (weeks)	34.6 (2.6)	39.2 (1.3)	<0.0001	36.5 (3.2)	39.1 (1.6)	0.0001	37.0 (3.2)	38.4 (2.8)	0.006
Characteristics at study visit									
Study visit timing ^†^	33.3 (3.2)	33.0 (3.8)	0.68	16.9 (12.3)	9.7 (5.6)	0.002	6.0 (3.5)	5.9 (3.8)	0.88
Age (years)	33.4 (5.5)	32.9 (4.4)	0.65	34.3 (4.4)	33.5 (3.7)	0.41	40.6 (6.3)	41.0 (5.3)	0.54
Parity	0.8 (1.1)	0.4 (0.5)	0.05	1.6 (1.0)	1.4 (0.5)	0.21	2 (1.2)	2 (0.8)	0.63
Nulliparous/primiparous ^‡^	19 (59%)	25 (60%)	0.99	28 (64%)	23 (64%)	0.98	26 (37%)	22 (30%)	0.35
Gravidity	2.6 (1.6)	1.8 (0.8)	0.005	2.3 (1.3)	1.8 (0.8)	0.04	3 (1.9)	3 (1.6)	0.52
Body mass index (kg/m^2^)	33.2 (6.8)	26.2 (3.6)	<0.0001	28.5 (6.6)	24.2 (3.6)	0.002	27 (4.9)	24 (4.9)	0.005
Blood pressure ^§^									
Systolic	132 (14.6)	105 (7.0)	<0.0001	115 (9.1)	106 (7.9)	0.0006	119 (10.7)	112 (11.5)	0.0003
Diastolic	82 (5.9)	64 (6.7)	<0.0001	73 (7.3)	68 (5.9)	0.005	75 (8.5)	70 (9.3)	0.001
Antihypertensive treatment ^||^	9 (28%)	0 (0%)	0.0002	2 (5%)	0 (0%)	0.21	7(10%)	2 (3%)	0.006

Data are presented as either number (percentage) or mean (standard deviation). * Participants could indicate more than one race, so totals are >100%; ^†^ in relation to antepartum, gestational age in weeks; ^†^ in relation to postpartum, number of weeks post-delivery; ^†^ in relation to mid-life, number of years since last delivery, ^‡^ in relation to antepartum, proportion who were nulliparous at the antepartum visit; ^‡^ in relation to postpartum and mid-life, proportions who were primiparous at the study visits; ^§^ average of last three blood pressures measured at the time of the visit; ^||^ within the 48 h prior to the study visit.

**Table 2:** Metabolites that differed most significantly between cases and controls antepartum.

Metabolite Name	Class	Pathway	Log2 (Fold-Change)	Adjusted *p*-Value
10,20-Dihydroxyeicosanoic acid *	Eicosanoid and resolvin	Eicosanoid and resolvin metabolism	−2.05	0.00001
Pregnenolone sulfate	Gonadal steroid	Gonadal steroid metabolism/xenobiotic metabolism	−1.13	0.00003
Cortisol	Steroids and derivatives	Cholesterol, cortisol, non-gonadal steroid metabolism	−1.98	0.00003
11β-Hydroxyprogesterone	Steroids and derivatives	Gonadal steroid metabolism	−2.01	0.00021
Butyrylcarnitine	Acyl carnitines	Fatty Acid Oxidation and Synthesis	0.84	0.00026
L-Tyrosine	Amino acids and derivatives	Tyrosine and phenylalanine metabolism	0.42	0.00026
Estrone 3-sulfate	Steroids and derivatives	Gonadal steroid metabolism/xenobiotic metabolism	−1.81	0.00027
Hydroxyoctadienoic acid	Lipid and lipid-like	Fatty acid oxidation and synthesis	0.72	0.00030
C7H15N3O2	NA	NA	0.87	0.00030
Pregnanolone sulfate	Steroids and derivatives	Gonadal steroid metabolism/xenobiotic metabolism	−0.67	0.00032
C17H27NO4	NA	NA	0.87	0.00033
Estriol-3-glucuronide	Steroids and derivatives	Gonadal steroid metabolism/xenobiotic metabolism	−1.74	0.00033
Dihydrocortisol **	Steroids and derivatives	Cholesterol, cortisol, non-gonadal steroid Metabolism	−2.38	0.00034
Hydroxyestrone sulfate	Steroids and derivatives	Gonadal steroid metabolism/xenobiotic metabolism	−1.70	0.00034
9-Nor-1-,25-dihydroxyvitamin D2	Vitamins and derivatives	Vitamin metabolism	−0.68	0.00037
L-Phenylalanine	Amino acids and derivatives	Tyrosine and phenylalanine metabolism	0.30	0.00047
Gamma-glutamyl-L-isoleucine	Dipeptide	Protein digestion or protein catabolism	0.46	0.00047
Glutamylphenylalanine	Dipeptide	Protein digestion or protein catabolism	0.46	0.00092

* Significant independent predictor in the antepartum and postpartum multivariate models ** Significant independent predictor in the antepartum multivariate model.

## Data Availability

The deidentified data used in this study, a data dictionary, and the code used to analyze it will be available on the Zenodo repository.

## Supplementary Materials

The following supporting information can be downloaded at: https://www.mdpi.com/article/10.3390/ijms26136150/s1.
