# Supplementary material for: Metabolomic Profiles During and After a Hypertensive Disorder of Pregnancy: The EPOCH Study
Source: Int J Mol Sci. 2025 Jun 26;26(13):6150. doi: 10.3390/ijms26136150 (PMC12250391; doi:10.3390/ijms26136150)
Supplement: Supplementary file 1 [file ijms-26-06150-s001.zip › ijms-3648376-supplementary.pdf]

Supplementary Figure 1:
Effect of betamethasone (BMZ) treatment on corticosteroid levels among cases

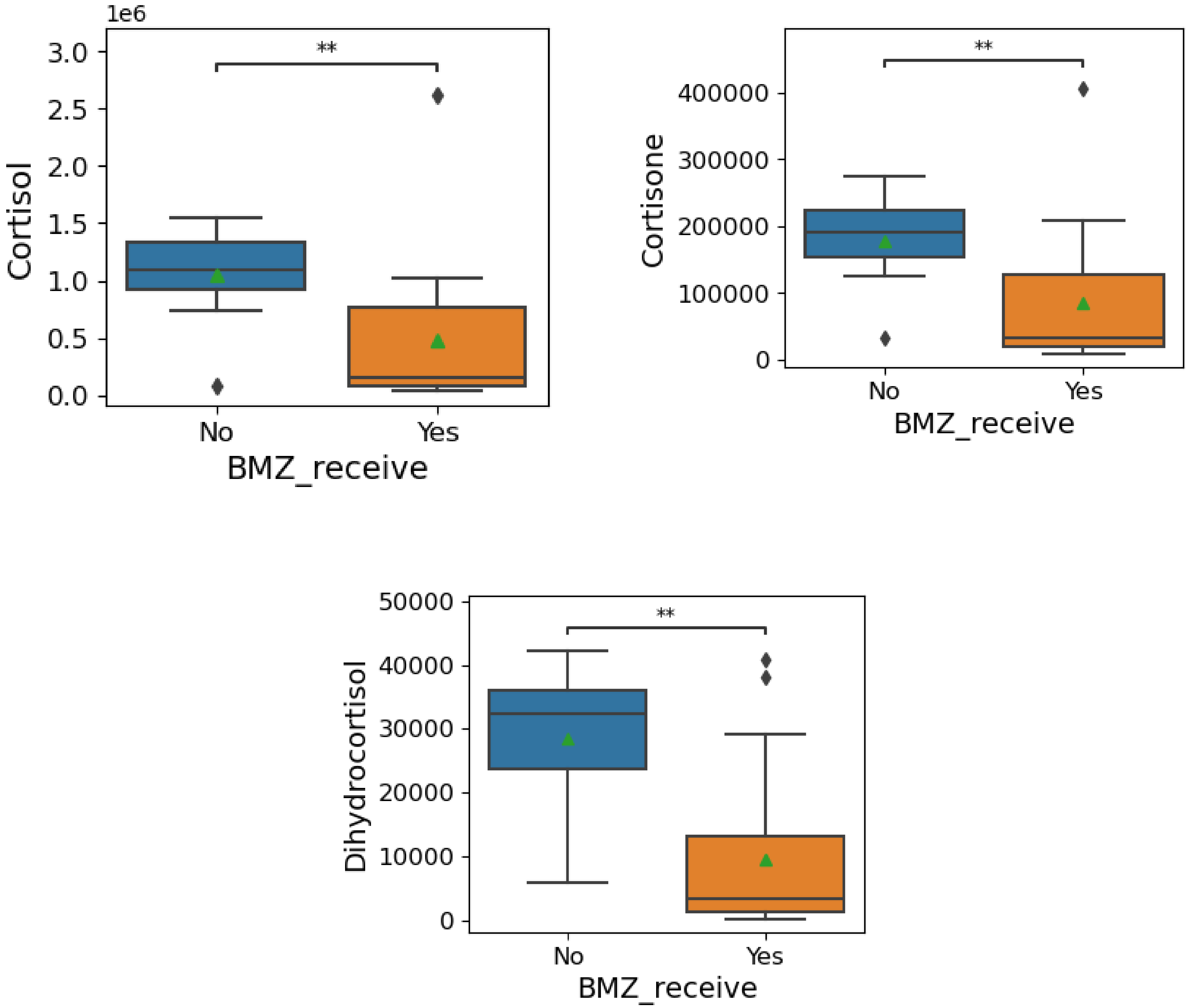

Supplementary Figure 2.
Timing of betamethasone treatment (vertical axis) and plasma corticosteroid levels (horizontal axis).

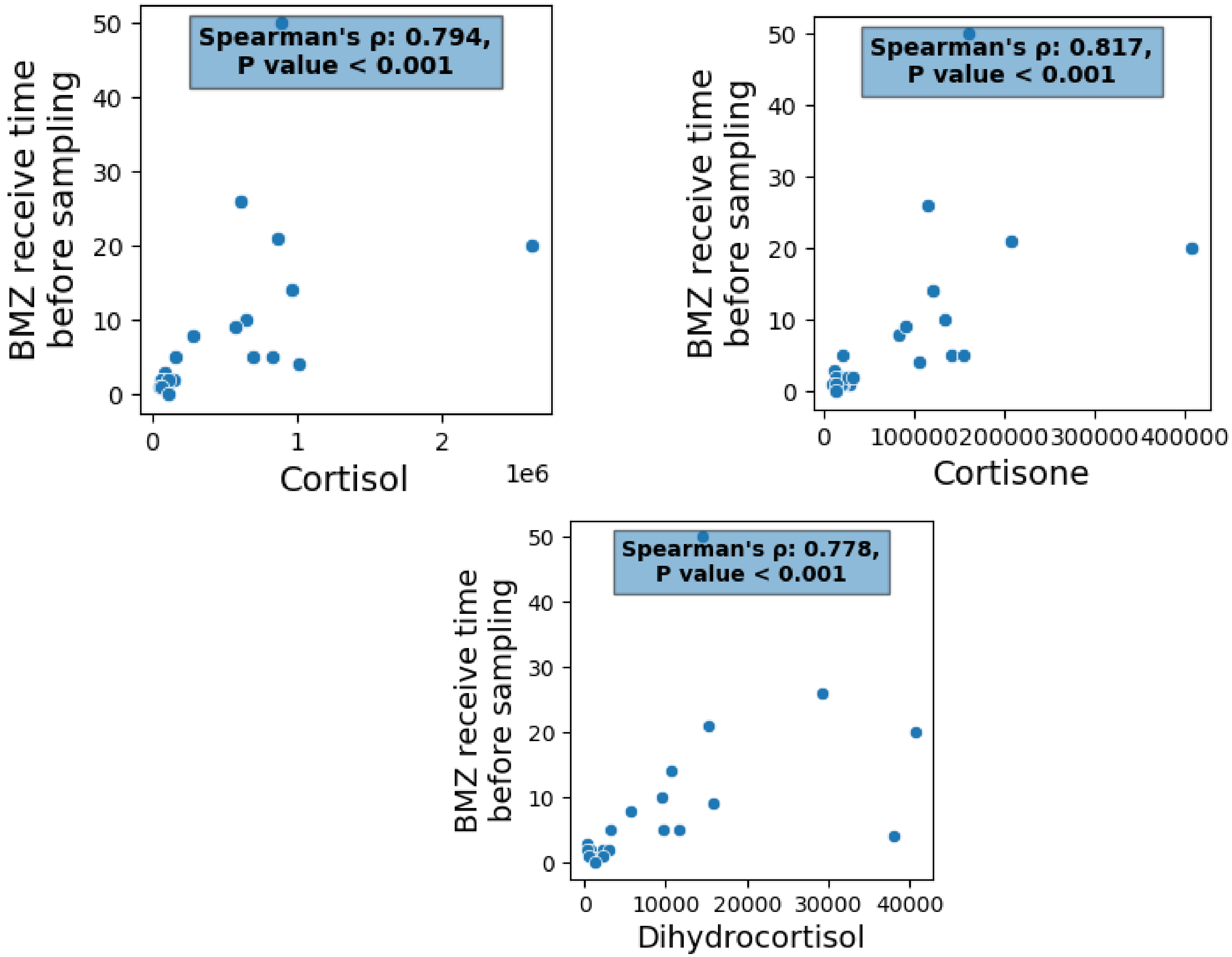

name_short	class	pathway	log2 Fold-change	Adjusted p-value
10,20-Dihydroxyeicosanoic acid	Eicosanoid and Resolvin	Eicosanoid and Resolvin Metabolism	-2.0478	0.00001
Pregnenolone sulfate	Steroids and derivatives	Progestin Steroids/Xenobiotic Metabolism	-1.1315	0.00003
Cortisol	Steroids and derivatives	Cholesterol, Cortisol, Non-Gonadal Steroid Metabol	-1.9752	0.00003
11b-Hydroxyprogesterone	Steroids and derivatives	Gonadal Steroid Metabolism	-2.0093	0.00021
L-Tyrosine	Amino acids and derivatives	Tyrosine and Phenylalanine Metabolism	0.4175	0.00026
Butyrylcarnitine	Acyl carnitines	Microbiome Metabolism	0.8443	0.00026
estrone 3-sulfate	Steroids and derivatives	Progestin Steroids/Xenobiotic Metabolism	-1.8099	0.00027
apo-[3-methylcrotonoyl-CoA:carbon-d	Organic acids and derivatives	Other	0.8671	0.00030
Hydroxy-octadienoic acid	Lipid and lipid like	Fatty Acid Oxidation and Synthesis	0.7244	0.00030
Pregnanolone sulfate	Steroids and derivatives	Progestin Steroids/Xenobiotic Metabolism	-0.6653	0.00032
NA	NA	Other	0.8667	0.00033
Estriol-3-glucuronide	Steroids and derivatives	Progestin Steroids/Xenobiotic Metabolism	-1.7382	0.00033
Dihydrocortisol	Steroids and derivatives	Cholesterol, Cortisol, Non-Gonadal Steroid Metabol	-2.3842	0.00034
Hydroxyestrone sulfate	Steroids and derivatives	Progestin Steroids/Xenobiotic Metabolism	-1.7028	0.00034
9-Nor-1-,25-dihydroxyvitamin D2	Vitamins and derivatives	Vitamin Metabolism	-0.6808	0.00037
L-Phenylalanine	Amino acids and derivatives	Tyrosine and Phenylalanine Metabolism	0.3012	0.00047
gamma-glutamyl-L-isoleucine	Depeptide	Protein digestion or protein catabolism	0.4562	0.00047
Glutamylphenylalanine	Depeptide	Protein digestion or protein catabolism	0.4608	0.00092
Cyanuric acid	NA	Other	0.5182	0.00106
Indole-3-propionic acid(IPA)	Indoles and derivatives	Tryptophan, Kynurenine, Serotonin, Melatonin Meta	-1.3397	0.00117
LysoPE(20:4)	lipid and lipid like	Glycerophospholipid Metabolism (PUSFA)	0.1366	0.00143
N-Phenylglycine	Amino acids and derivatives	Other	1.7748	0.00159
Octenoylcarnitine	Acyl carnitines	Fatty Acid Oxidation and Synthesis_Acyl Carnitine	0.9028	0.00159

Isovalerylcarnitine	Acyl carnitines	Branch Chain Amino Acid Metabolism	0.5871	0.00161
Cortisone	Steroids and derivatives	Cholesterol, Cortisol, Non-Gonadal Steroid Metabol	-1.5196	0.00161
Uric acid	Nucleotides and derivatives	Purine Metabolism	0.5558	0.00252
1,24,25-trihydroxyvitamin D3	Vitamins and derivatives	Vitamin Metabolism	-0.3994	0.00270
3-Indoxyl sulfate	Organic acids and derivatives	Tryptophan, Kynurenine, Serotonin, Melatonin Meta	3.0410	0.00270
LysoPE(18:0)	lipid and lipid like	Glycerophospholipid Metabolism (SFA)	0.6200	0.00273
Uric acid	Nucleotides and derivatives	Purine Metabolism	0.4849	0.00290
Tetradecanedioic acid	Lipid and lipid like	Fatty Acid Oxidation and Synthesis	-0.9116	0.00294
Estriol sulfate glucuronide	Steroids and derivatives	Progestin Steroids/Xenobiotic Metabolism	-1.5732	0.00328
Cys-Pro or Pro-Cys	Depeptide	Protein digestion or protein catabolism	-0.9596	0.00329
gamma-glutamyl-L-isoleucine	Depeptide	Protein digestion or protein catabolism	0.4929	0.00329
Xanthine	Nucleotides and derivatives	Purine Metabolism	0.4531	0.00329
Androstane-3,17-diol 17-sulfate	Steroids and derivatives	Gonadal Steroid Metabolism/Xenobiotic Metabolism	-0.8338	0.00335
LysoPE(22:5)	lipid and lipid like	Glycerophospholipid Metabolism (PUSFA)	0.7323	0.00341
N-Phenylglycine	Amino acids and derivatives	Other	2.8462	0.00348
gamma-glutamyl-epsilon-lysine	Organic acids and derivatives	Amino acid Metabolism	1.5444	0.00389
17-Hydroxypregnenolone 3-sulfate	Steroids and derivatives	Progestin Steroids/Xenobiotic Metabolism	-0.7784	0.00397
Butyrylcarnitine	Acyl carnitines	Microbiome Metabolism	0.5550	0.00414
Adrenic acid	Lipid and lipid like	Fatty Acid Oxidation and Synthesis	0.9750	0.00414
Glutamylphenylalanine	Amino acids and derivatives	Tyrosine and Phenylalanine Metabolism	0.4876	0.00423
LysoPI(20:4)	Lipid and lipid like	Glycerophospholipid Metabolism (PUSFA)	0.5566	0.00433
3-Indolepropionic acid (IPA)	Indoles and derivatives	Tryptophan, Kynurenine, Serotonin, Melatonin Meta	-1.0774	0.00433
gamma-CEHC glucuronide	Carbohydrates and carbohydra	Carbohydrates Metabolism	0.7497	0.00433
L-Palmitoylcarnitine	Acyl carnitines	Fatty Acid Oxidation and Synthesis_Acyl Carnitine	0.4766	0.00475

2-trans,4-cis-Decadienoylcarnitine	Acyl carnitines	Fatty Acid Oxidation and Synthesis_Acyl Carnitine	0.9442	0.00475
L-isoleucyl-L-proline	Depeptide	Protein digestion or protein catabolism	0.7449	0.00486
25-Hydroxyvitamin D3-26,23-lactol	Vitamins and derivatives	Vitamin Metabolism	-0.5931	0.00488
LysoPI(18:1)	Lipid and lipid like	Glycerophospholipid Metabolism (MUSFA)	0.7017	0.00488
2-Acetolactate	Organic acids and derivatives	TCA Cycle	0.3121	0.00513
Pregnenolone	Steroids and derivatives	Progestin Steroids	0.7777	0.00513
8-Aminooctanoic acid	Lipid and lipid like	Fatty Acid Oxidation and Synthesis	0.4954	0.00527
Trihydroxycholestanoic acid	Lipid and lipid like	Bile acid Metabolism	-0.4786	0.00590
Phenylalanine	Amino acids and derivatives	Tyrosine and Phenylalanine Metabolism	0.2312	0.00610
glycodeoxycholate sulfate	Steroids and derivatives	Gonadal Steroid Metabolism/Xenobiotic Metabolism	-0.9269	0.00610
LysoPE(18:0)	Lipid and lipid like	Glycerophospholipid Metabolism (SFA)	0.5496	0.00610
NA	NA	Other	0.7248	0.00643
Taurocholic acid	Lipid and lipid like	Bile acid Metabolism	1.9099	0.00643
Isovalerylcarnitine	Acyl carnitines	Branch Chain Amino Acid Metabolism	0.4210	0.00690
LysoPE(P-16:0)	Lipid and lipid like	Glycerophospholipid Metabolism (SFA)	0.4306	0.00709
N-Methyl-L-threonine	Amino acids and derivatives	Amino acid Metabolism	-0.4053	0.00728
Hydroxybutyrylcarnitine	Acyl carnitines	Microbiome Metabolism	0.8174	0.00748
Hydroxyandrosterone-3-glucuronide	Steroids and derivatives	Gonadal Steroid Metabolism/Xenobiotic Metabolism	-1.2258	0.00802
5-Methoxyindole	Indoles and derivatives	Microbiome Metabolism	-1.0205	0.00834
Propionylcarnitine	Acyl carnitines	Fatty Acid Oxidation and Synthesis_Acyl Carnitine	0.8232	0.00834
LysoPE(18:0)	lipid and lipid like	Glycerophospholipid Metabolism (SFA)	0.6319	0.00858
6-Benzylaminopurine	NA	Other	-0.6874	0.00882
Dopamine quinone (DoQ)	Neurotransmitter	Tyrosine and Phenylalanine Metabolism	1.9678	0.00906
Tiglylcarnitine	Acyl carnitines	Branch Chain Amino Acid Metabolism	0.4841	0.00932

Formiminoglutamic acid	Amino acids and derivatives	GABA, Glutamate, Arginine, Ornithine, Proline Meta	0.4801	0.00958
phenylacetylserine	Amino acids and derivatives	Amino acid Metabolism	-0.9507	0.01027
Hovenolactone	NA	Other	-1.8853	0.01101
Undecanedioic acid	lipid and lipid like	Fatty Acid Oxidation and Synthesis	0.4143	0.01229
Octenoylcarnitine	Acyl carnitines	Fatty Acid Oxidation and Synthesis_Acyl Carnitine	0.7244	0.01264
Methyladipic acid	Lipid and lipid like	Fatty Acid Oxidation and Synthesis	0.3721	0.01409
N-methylproline	Amino acids and derivatives	GABA, Glutamate, Arginine, Ornithine, Proline Meta	-1.0143	0.01490
Dehydroisoandrosterone sulfate (DHEA	Steroids and derivatives	Cholesterol, Cortisol, Non-Gonadal Steroid Metabol	-1.3249	0.01490
Phosphatidylglycerol	lipid and lipid like	Glycerophospholipid Metabolism	-0.3437	0.01531
Tauroursodeoxycholic acid	Lipid and lipid like	Bile acid Metabolism	1.3475	0.01639
Adenosine	Nucleotides and derivatives	Purine Metabolism	0.5066	0.01753
LysoPE(18:2)	lipid and lipid like	Glycerophospholipid Metabolism (PUSFA)	0.1589	0.01760
L-Carnitine	Acyl carnitines	Fatty Acid Oxidation and Synthesis_Acyl Carnitine	0.5183	0.01760
Hexenoylcarnitine	Acyl carnitines	Fatty Acid Oxidation and Synthesis_Acyl Carnitine	0.6799	0.01760
NA	NA	Other	-0.9598	0.01769
Trihydroxy-5beta-cholestan-27-al	Lipid and lipid like	Cholesterol, Cortisol, Non-Gonadal Steroid Metabol	-0.7104	0.01769
NA	NA	Other	0.3405	0.01769
Trihydroxy-5beta-cholestan-27-al	Lipid and lipid like	Cholesterol, Cortisol, Non-Gonadal Steroid Metabol	-0.7039	0.01800
Histidine	Nucleotides and derivatives	Histidine, Histamine, Carnosine Metabolism	-0.6252	0.01800
1,25-Dihydroxy-16-ene-vitamin D3	Vitamins and derivatives	Vitamin Metabolism	-0.4485	0.01832
Hydroxyglutaric acid	Organic acids and derivatives	TCA Cycle	0.3210	0.01832
LysoPC(22:5)	lipid and lipid like	Glycerophospholipid Metabolism (PUSFA)	0.6360	0.02074
N6-Carbamoyl-L-threonyladenosine	Nucleotides and derivatives	Purine Metabolism	0.2157	0.02074
L-Acetylcarnitine	Acyl carnitines	Fatty Acid Oxidation and Synthesis_Acyl Carnitine	0.5979	0.02074

NA	NA	Other	0.1654	0.02112
Hydroxy-octadienoic acid	Lipid and lipid like	Fatty Acid Oxidation and Synthesis	0.1849	0.02112
Docosapentaenoic acid	Lipid and lipid like	Fatty Acid Oxidation and Synthesis	0.8326	0.02258
NA	NA	Other	0.3935	0.02323
NA	NA	Other	-0.5778	0.02367
NA	NA	Other	-0.4779	0.02367
L-Isoleucine|L-Leucine	Amino acids and derivatives	Branch Chain Amino Acid Metabolism	0.3340	0.02411
Trimethyl-2-(1-methylethyl)butanamide	lipid and lipid like	Other	0.2534	0.02411
Methionine sulfoxide	Amino acids and derivatives	Methionine, Cysteine, SAM and Taurine Metabolism	0.4498	0.02481
Methionine sulfoxide	Amino acids and derivatives	Methionine, Cysteine, SAM and Taurine Metabolism	0.4498	0.02481
NA	NA	Other	-1.3201	0.02505
N6-Carbamoyl-L-threonyladenosine	Nucleotides and derivatives	Purine Metabolism	0.2579	0.02505
4-Hydroxyphenylacetic acid sulfate	Benzenoids	Xenobiotic Metabolism	2.0293	0.02505
Hydroxy-3-oxotetradecenoic acid	Lipid and lipid like	Fatty Acid Oxidation and Synthesis	-0.5210	0.02555
Edetic Acid	NA	drug	0.2407	0.02555
Armillane	NA	Other	-1.5279	0.02560
1,24,25-trihydroxyvitamin D3	Vitamins and derivatives	Vitamin Metabolism	-1.0599	0.02560
NA	NA	Other	-0.6126	0.02560
N-(1-Deoxy-1-fructosyl)phenylalanine	Amino acids and derivatives	Other	0.5828	0.02560
Hydroxyhexanoic acid	lipid and lipid like	Fatty Acid Oxidation and Synthesis	0.2942	0.02635
5-HETrE	Eicosanoid and Resolvin	Eicosanoid and Resolvin Metabolism	0.4715	0.02712
Hydroxydecanoyl carnitine	Acyl carnitines	Fatty Acid Oxidation and Synthesis_Acyl Carnitine	0.7914	0.02897
17-Hydroxypregnenolone 3-sulfate	Steroids and derivatives	Progestin Steroids/Xenobiotic Metabolism	-0.6955	0.02981
MG(16:1)	Lipid and lipid like	Glycerolipid Metabolism	-0.7995	0.03017

11-Dodecenoic acid	lipid and lipid like	Fatty Acid Oxidation and Synthesis	-0.0571	0.03017
LysoPE(22:5)	Lipid and lipid like	Glycerophospholipid Metabolism (PUSFA)	0.5731	0.03017
5-Methylnicotinamide	Vitamins and derivatives	Vitamin B3 (Niacin, NAD+) Metabolism	-0.8627	0.03080
3-Hydroxynona-4,6-dienoylcarnitine	Acyl carnitines	Fatty Acid Oxidation and Synthesis_Acyl Carnitine	0.2500	0.03080
Threonylisoleucine	Depeptide	Protein digestion or protein catabolism	0.7161	0.03169
NA	NA	Other	-1.3161	0.03330
24R,25-Dihydroxyvitamin D3	Vitamins and derivatives	Vitamin Metabolism	-0.4846	0.03330
Stearoylcarnitine	Acyl carnitines	Fatty Acid Oxidation and Synthesis_Acyl Carnitine	0.7030	0.03330
19-Nor-1-,25-dihydroxyvitamin D2	Vitamins and derivatives	Vitamin Metabolism	-0.3844	0.03427
Dihydrocortisol	Steroids and derivatives	Cholesterol, Cortisol, Non-Gonadal Steroid Metabol	-1.8995	0.03499
Phosphatidylglycerol	lipid and lipid like	Glycerophospholipid Metabolism	-0.5789	0.03499
NA	NA	Other	-0.5425	0.03546
Trihydroxy-5beta-cholestan-27-al	Lipid and lipid like	Bile acid Metabolism	-0.3896	0.03546
L-Glutamine	Amino acids and derivatives	Bioamines and Neurotransmitter Metabolism	0.2378	0.03546
C11H18N2O2	NA	Other	0.5574	0.03701
Oleoylcarnitine	Acyl carnitines	Fatty Acid Oxidation and Synthesis_Acyl Carnitine	0.6387	0.03701
NA	NA	Other	0.3872	0.03701
Dihomo-alpha-linolenic acid	Lipid and lipid like	Eicosanoid and Resolvin Metabolism	0.6193	0.03701
Dehydroisoandrosterone sulfate (DHEA	Steroids and derivatives	Cholesterol, Cortisol, Non-Gonadal Steroid Metabol	-0.7209	0.03863
Pregnanolone sulfate	Steroids and derivatives	Progestin Steroids/Xenobiotic Metabolism	-0.6207	0.03863
Hydroxysebacic acid	Lipid and lipid like	Fatty Acid Oxidation and Synthesis	0.1577	0.03863
NA	NA	Other	0.3794	0.04091
Hydroxyoctanoic acid	Lipid and lipid like	Fatty Acid Oxidation and Synthesis	0.8684	0.04091
6-Benzylaminopurine	Nucleotides and derivatives	Purine Metabolism	-0.5088	0.04331

apo-[3-methylcrotonoyl-CoA:carbon-d	NA	Other	-0.2228	0.04331
LysoPC(20:3)	lipid and lipid like	Glycerophospholipid Metabolism (PUSFA)	0.5420	0.04395
7-Ketodeoxycholic acid	Lipid and lipid like	Bile acid Metabolism	-1.1219	0.04395
5-Hydroxy-2-imino-1-methylimidazolic	NA	Other	0.3334	0.04395
LysoPE(16:0)	Lipid and lipid like	Glycerophospholipid Metabolism (SFA)	-0.3679	0.04522
Oxo-androsterone glucuronide	Steroids and derivatives	Gonadal Steroid Metabolism/Xenobiotic Metabolism	-0.9742	0.04621
Glycerophosphocholine	Lipid and lipid like	Glycerophospholipid Metabolism	0.9375	0.04621
Malonylcarnitine	Acyl carnitines	Fatty Acid Oxidation and Synthesis_Acyl Carnitine	0.7567	0.04859
Pregnandiol monosulfate	Steroids and derivatives	Progestin Steroids/Oxidoreduction	-0.4888	0.04859
5-methyluridine (ribothymidine)	Nucleotides and derivatives	Pyrimidine Metabolism, Uracil containing	0.2304	0.04859
Tetrahydrocortisone glucuronide	Steroids and derivatives	Cholesterol, Cortisol, Non-Gonadal Steroid Metabol	-1.4930	0.04934
Hydroxy-pentadecanoic acid	Lipid and lipid like	Fatty Acid Oxidation and Synthesis	-0.5340	0.04934
L-Lysine	Amino acids and derivatives	Lysine Metabolism	0.2723	0.04934
